# MiR-876-5p modulates head and neck squamous cell carcinoma metastasis and invasion by targeting vimentin

**DOI:** 10.1186/s12935-018-0619-7

**Published:** 2018-08-28

**Authors:** Yibo Dong, Yang Zheng, Chundi Wang, Xu Ding, Yifei Du, Laikui Liu, Wei Zhang, Wei Zhang, Yi Zhong, Yunong Wu, Xiaomeng Song

**Affiliations:** 1 Jiangsu Key Laboratory of Oral Diseases, Nanjing Medical University, 140, Hanzhong Road, Nanjing, 210029 China; 2 Department of Oral and Maxillofacial Surgery, Affiliated Hospital of Stomatology, Nanjing Medical University, 136, Hanzhong Road, Nanjing, 210029 China; 3 Department of Oral Pathology, Affiliated Hospital of Stomatology, Nanjing Medical University, 136, Hanzhong Road, Nanjing, 210029 China

**Keywords:** miR-876-5p, HNSCC, Vimentin, Invasion, Metastasis

## Abstract

**Background:**

Local or distant metastasis remains the main course of death in head and neck squamous cell carcinoma (HNSCC) patients. MicroRNAs (miRNAs) have been implicated in metastasis of HNSCC, but the mechanisms of their action are mainly undocumented. Through public head and neck cancer miRNA expression datasets, we found that miR-876-5p was a novel potential tumor suppressor targeting HNSCC metastasis.

**Methods:**

Clinical significance and mechanism of miR-876-5P was systematically analyzed in HNSCC. Quantitative RT-PCR was used to evaluate miR-876-5p levels in HNSCC cell lines and in 20 pairs of HNSCC with associated regional nodal metastases and HNSCC without metastatic primary tumors. Scratch and invasion assays were evaluated to determine the role of miR-876-5p in the regulation of HNSCC cell migration and invasion, respectively. Western blotting was used to investigate the mechanism by which miR-876-5p suppresses HNSCC cell invasion and migration. Luciferase assays were performed to assess miR-876-5p binding to the vimentin gene. The animal model was used to support the in vitro experimental findings.

**Results:**

MiR-876-5p mimics inhibited HNSCC cell migration and invasion. Vimentin protein and mRNA levels were decreased in the miR-876-5p mimics group but increased in the miR-876-5p inhibitors group, which demonstrated that miR-876-5p inhibits vimentin expression in HNSCC cells. By directly targeting the vimentin 3′-UTR, we used dual-luciferase reporter assays to verify that vimentin is a functional downstream target of miR-876-5p. Importantly, increased vimentin expression promoted cell migration and invasion, and co-transfection with miR-876-5p mimics and vimentin restored cell aggressiveness to the original level. Moreover, miR-876-5p overexpression significantly downregulated vimentin expression level and inhibited the distal metastasis of HNSCC cells in vivo.

**Conclusions:**

miR-876-5p, which functions as a tumor suppressor in HNSCC, inhibits metastasis by targeting vimentin and provides a novel therapeutic target for HNSCC treatment.

**Electronic supplementary material:**

The online version of this article (10.1186/s12935-018-0619-7) contains supplementary material, which is available to authorized users.

## Background

Head and neck squamous cell carcinoma (HNSCC) is the sixth most common cancer in the world [1]. More than half of the newly diagnosed HNSCC patients have been reported to suffer from advanced-stage disease, and the majority of them may cause the spread of the tumor into the cervical regional draining lymph nodes [2]. It is clinically relevant to assess whether a patient has, or will develop, regional lymph node metastases [3]. Therefore, it is necessary to obtain deeper understanding of HNSCC metastasis and to develop predictive molecular signatures, which could improve survival rate and guide the development and evaluation of new therapies [4].

MicroRNAs (miRNAs) are endogenous, small, non-coding, and single-stranded RNAs (approximately 22 nucleotides in length) which can inhibit the expression of target genes at the post-transcriptional level by binding to the 3′-untranslated region (3′-UTR) of mRNAs, resulting in translation inhibition or mRNA degradation [5–7]. A growing number of miRNAs have been observed in head and neck cancers and shown to regulate the biological behavior of cancer cells [8]. It has been reported that downregulation of let-7a was associated with the presence of perineural invasion and upregulation of miR-145 and miR-205 in HNSCC samples was associated with the existence of vascular invasion and lymph node metastasis, respectively [9]. By using fine-needle aspiration (FNA) biopsies of HNSCC patients, miR-203 and miR-205 were demonstrated in all metastatic samples, regardless of the size of the metastatic deposit [10]. However, there are still few investigations related to tumor suppressor microRNAs associated with lymph node metastases.

MiR-876-5p is a newly discovered miRNA which was reported to be linked to the acquisition of metastatic properties in bladder cancers [11]. It was also reported as a tumor suppressor in hepatocellular carcinoma [12], colorectal cancer [13], gastric cancer [14] and Hodgkin lymphoma [15]. It was recently shown that miR-876-5p can suppress epithelial–mesenchymal transition (EMT) by directly down-regulating bone morphogenetic protein 4 in lung cancer [16]. However, whether miR-876-5p exerts an effect on tumorigenesis and metastasis of HNSCC remains unknown.

Tumor recurrence and metastasis usually result in a poor prognosis of HNSCC patients [17]. The process of metastasis is very complicated and is known as a late incident in tumorigenesis [18]. Cells losing contact with adjacent cells, migrate through the interstitial matrix, invade blood and lymph vessels, and develop again in lymph nodes [19]. The metastatic cells therefore have to possess several properties to be able to perform all of these actions. The metastatic behavior of a tumor will be based on overexpression of metastasis promoting factors and loss of expression of suppressing factors [20, 21]. EMT is a process that is currently in the limelight of investigating the onset of cancer cell migration, invasion and metastatic dissemination [17]. Vimentin is an intermediate-sized filament that is highly expressed in mesenchymal cells and is generally used to identify cancer cells proceeding EMT based on a positive correlation of vimentin expression with increased invasiveness and metastasis [22]. Its expression in oral epithelial cells has been pathologically associated with tumor invasion and metastasis [23].

In this study, we first confirmed the anti-metastatic effect of miR-876-5p on HNSCC. The expression of miR-876-5p in HNSCC tissues and cell lines was detected by qRT-PCR. Its inhibitory roles of against cell migration and metastasis in HNSCC were confirmed in vivo and in vitro. By using bio-informatics analysis (Target Scan) and experimentally validation, we found that vimentin was a direct-target of miR-876-5p. MiR-876-5p can inhibit HNSCC metastasis and EMT by targeting vimentin, revealing its correlation with the prognosis of metastatic HNSCC.

## Methods

### Cell culture, plasmids, and transfection

The CAL27 and HEK293T (293T) cell lines were obtained from the American Type Culture Collection (ATCC). Human HNSCC WSU-HN4 and WSU-HN6 (hereafter simplified to HN4 and HN6) cell lines were obtained from Dr. Silvio Gutkind of the NIH (Bethesda, MD) [24, 25]. CAL27 cells (authenticated at July 2017) and HEK293T cells (authenticated at March 2018) were tested by short tandem repeat testing done in the Fragment Analysis Facility, Tian Lin Biology and Technology Company (Shanghai, China). The two cell lines utilized in the experiment (WSU-HN4, WSH-HN6) showed distinct genotypes not matching with any in the databases. All the cell lines were recovered and maintained in Dulbecco’s modified Eagle’s medium containing 10% FBS at 37 °C in an atmosphere of 5% CO_2_. The hsa-miR-876-5p mimics, the hsa-miR-876-5p inhibitors and corresponding negative controls were purchased from GenePharma (Shanghai, China). The RNA sequences for the primers mentioned above were as follows: miR-876-5p mimics, sense 5′-UGGAUUUCUUUGUGAAUCACCA-3′ and antisense 5′-GUGAUUCACAAAGAAAUCCAUU-3′; mimic control, sense 5′-UUCUCCGAACGUGUCACGUTT-3′ and antisense 5′-ACGUGACACGUUCGGAGAATT-3′; miR-876-5p inhibitors 5′-UGGUGAUUCACAAAGAAAUCCA-3′; and inhibitor control 5′-CAGUACUUUUGUGUAGUACAA-3′. The vimentin overexpression and negative control plasmids were obtained from GeneCopoeia (EX-vimentin-M02 and EX-EGFP-M02, respectively). The CAL27 and HN6 cells were transfected with special vimentin overexpression and negative control plasmids for 8 h using Lipofectamine 2000 (Invitrogen, USA) following the manufacturer’s instructions. The efficacy of transfection was tested by qRT-PCR and western blotting. For the manual alteration of miR-876-5p expression, cells (5 × 10^5^ per well in 6-well plates) were cultured to 80% confluence in complete growth medium; then, the medium was replaced with serum-free medium with miRNA mimics or inhibitors using Lipofectamine 2000 for 4–6 h according to the manufacturer’s instructions.

### Patients and sample collection

A total of 40 primary HNSCC cases were histopathologically and clinically diagnosed in the Stomatological Hospital of Jiangsu Province, Nanjing Medical University between 2017 and 2018. The details of patients were presented in Table 1. None of the patients had been treated with any tumor-specific therapy before surgery. Tissue samples from HNSCC patients without metastatic primary tumors were collected during the study period and served as the normal controls for comparison with the tissue samples from HNSCC patients with associated regional nodal metastases. After collection, all tissues samples were immediately frozen in liquid nitrogen and stored at − 80 °C until further use. All tissues were obtained with patient consent, and written informed consent for the use of tissue samples for research purposes was obtained from the donors or the next of kin. This study was approved by the institutional ethics committee of the Nanjing Medical University.

**Table 1:** Clinical features of 40 patients with HNSCC

No.	Age	Sex	Location	T	N	M	Differentiation
1	59	M	Gingiva	1	0	0	Well
2	69	F	Buccal	2	0	0	Well
3	64	F	Buccal	2	0	0	Well
4	61	M	Palate	2	0	0	Poor
5	65	M	Gingiva	3	0	0	Moderate
6	72	M	Buccal	2	0	0	Moderate
7	60	M	Oropharynx	2	0	0	Moderate
8	59	M	Palate	2	0	0	Well
9	64	F	Gingiva	4a	0	0	Poor
10	65	M	Buccal	2	0	0	Moderate
11	38	F	Tongue	2	0	0	Moderate
12	60	M	Tongue	4a	0	0	Poor
13	67	F	Tongue	4a	0	0	Moderate
14	79	F	Palate	4a	0	0	Poor
15	49	F	Gingiva	3	0	0	Poor
16	43	M	Gingiva	4a	0	0	Moderate
17	61	M	Buccal	2	0	0	Poor
18	54	M	Tongue	1	0	0	Moderate to well
19	81	M	Tongue	2	0	0	Moderate
20	68	F	Buccal	1	0	0	Moderate
21	50	M	Gingiva	2	1	0	Moderate
22	67	M	Buccal	2	1	0	Moderate
23	63	M	Tongue	3	1	0	Poor
24	66	M	Gingiva	4	2a	0	Poor
25	50	F	Tongue	2	1	0	Poor
26	78	F	Tongue	1	2b	0	Moderate
27	56	M	Buccal	2	1	0	Well
28	66	M	Tongue	3	2c	0	Well
29	54	F	Buccal	4a	2b	0	Well
30	56	M	Gingiva	4a	2b	0	Poor
31	78	M	Buccal	3	2b	0	Well
32	69	M	Gingiva	2	2b	0	Poor
33	68	F	Buccal	3	1	0	Moderate
34	74	F	Buccal	4a	2a	0	Moderate
35	57	M	Tongue	4a	1	0	Poor
36	67	M	Gingiva	4a	1	0	Moderate
37	54	F	Gingiva	4a	1	0	Moderate
38	72	M	Gingiva	3	1	0	Moderate
39	74	F	Gingiva	2	1	0	Poor
40	71	F	Tongue	3	1	0	Moderate

*HNSCC* head and neck squamous cell carcinoma, *F* female, *M* male; TNM classification and tumor stage were determined by the Union for International Cancer Control (UICC); HNSCC without metastatic primary tumors (No. 1–20); HNSCC with associated regional nodal metastases (No. 21–40)

### Real-time reverse transcription (RT)-PCR

Total RNA was extracted using a TRIzol reagent (Invitrogen) according to the manufacturer’s protocol. For qRT-PCR detection of mature miR-876-5p expression, we purchased the Bulge-Loop™ miRNA qRT-PCR Primer Set and Control Primer Set (RiboBio, Guangzhou, China). RNA (2 μg) was converted into cDNA using the RevertAid First Strand cDNA Synthesis Kit (Thermo). QRT-PCR was accomplished using the FastStart Universal SYBR Green Master Mix (Rox) (Roche) in the ABI PRISM^®^ 7300 real-time PCR system (Applied Biosystems, Foster City, CA, USA) according to the manufacturer’s instructions. GADPH and U6 were used as endogenous controls. We used dissociation curves to monitor non-specific amplification. The relative expression level was computed using the 2^−ΔΔCt^ method. The sequences for sense and antisense primers are as follows: vimentin 5′-TGA GTA CCG GAG ACA GGT GCA G-3′ (sense) and 5′-TAGCAG CTT CAA CGG CAA AGT TC-3′ (antisense) and GAPDH 5′-GAA GGT GAA GGT CGG AGT C-3′ (sense) and 5′-GAG ATG GTG ATG GGA TTT C-3′ (antisense). For miRNA quantification, Bulge-loop™ miRNA qRT-PCR Primer Sets (one RT primer and a pair of qPCR primers for each set) specific for U6 and miR-876-5p were designed by RiboBio (Guangzhou, China).

### Western blotting

Total protein was extracted from cells and lysed for 30 min using lysis buffer (Beyotime Shanghai, China). All proteins were resolved using sodium dodecyl sulfate–polyacrylamide gel electrophoresis (SDS-PAGE) with 10% polyacrylamide gels and then transferred to polyvinylidene difluoride (PVDF) membranes (Millipore, MA), which were blocked with 5% BSA in phosphate-buffered saline (PBS) containing Tween 20 (PBS-T) for 2 h at room temperature. The blots were then probed with primary antibodies specific for vimentin (1:1000; Proteintech, 60330-1-Ig, China) or beta-actin (1:1000; Bioworld, I102, China) overnight at 4 °C, washed twice with TBST, and incubated with horseradish peroxidase-conjugated (HRP) secondary antibodies (Zhongshan Golden Bridge Bio, China) for 1 h at room temperature. Finally, the protein bands were detected using Immobilon Western Chemiluminescent HRP substrate (Millipore) and visualized using the ImageQuantLAS 4000 mini imaging system (General Electrics).

### Invasion assays

Cell invasion ability was analyzed using Transwell filters (8 mm pore size; Millipore). Transwell inserts with 8-mm pores were coated with Matrigel (Matrigel:DMEM = 1:9; 50 µL per well; BD Bioscience, Franklin Lakes, NJ, USA). The cells (1 × 10^5^) were plated in 200 µL of serum-free medium in the upper chamber, while 500 µL of medium containing 10% FBS was used as the chemoattractant and placed into the lower chamber. After incubating the cells for 24 or 48 h at 37 °C, the non-invading cells remaining on the upper side of the filter were gently removed with cotton swabs. The invading cells on the lower membrane were fixed with 4% paraformaldehyde (PFA) for 30 min and stained with crystal violet for 5 min.

### Scratch assays

Cells were cultured to 90% confluence in 6-well plates and then scratched in the central area with a sterile 10-µL pipette tip. Floating cells and debris were carefully removed with PBS, and the culture medium was replaced with a serum-free medium. Wounded cell migration was observed under a microscope, and images of the same wound area were captured over time.

### Cell counting kit-8 (CCK-8) experiments

Cells were seeded in 96-well microplates at a density of 2 × 10^3^ cells per well. Cells were incubated in new medium containing 10% CCK-8 reaction solution (Selleckchem, Houston). After incubation for 2 h, the absorbance was measured on a spectrophotometer microplate reader (Multiskan MK3, Thermo) at a wavelength of 450 nm according to the manufacturer’s instructions. Three independent experiments were performed.

### Immunofluorescence staining

Briefly, HN6 and CAL27 cells were grown on cover slips for 24 h, and the cells were fixed in 4% PFA and permeabilized in 1% Triton. After incubating overnight with primary antibody against vimentin (1:100, Proteintech, 60330-1-Ig, China), the cells were incubated with FITC-conjugated ATF4 Rabbit Polyclonal antibody (1:500, Proteintech, FITC-10835, China) and counterstained with DAPI (Beyotime Shanghai, C1002, China). Cells were subsequently viewed by fluorescence microscopy (ZEISS, Germany).

### Immunohistochemistry

Tumor specimens were fixed in 10% neutral-buffered formalin for 24 h, followed by standard tissue processing and embedding. Sections were cut at 4 μm and dried overnight at 37 °C onto microscope slides. The tissue sections were stained with primary antibodies against vimentin (1:2000, Proteintech, 60330-1-Ig, China) and Ki67 (1:4000, Proteintech, 27309-1-AP, China) overnight following secondary antibody incubation for 30 min. All the sections were counterstained using hematoxylin and were dehydrated, cleared, and mounted before being examined using a microscope (DM4000B, Leica, Germany).

### Vector construction and dual-luciferase reporter assays

For luciferase assays, the potential miR-876-5p binding site in the vimentin 3′-UTR was predicted by TargetScan (http://www.targetscan.org) to be at position 47–56 from the vimentin stop codon site. All vimentin 3′-UTR sequences with the wild-type or mutant seed region were synthesized and cloned into the Pezx-FR02 vector (Genecopoeia, USA) downstream from the luciferase stop codon; the new vectors were designated vimentin-WT or vimentin-MUT, respectively. 293T cells were cotransfected with 50 nM miR-876-5p mimics or negative control and 1 µg of vimentin-WT or vimentin-MUT using Lipofectamine 2000 (Invitrogen, USA). Cells were harvested at 48 h after transfection, and luciferase activities were analyzed by the Luc-Pair Duo-Luciferase Assay Kit 2.0 (Genecopoeia, USA).

### Study in vivo

All animal studies were approved and supervised by the Animal Care and Use Committee of Nanjing Medical University, and all procedures were performed in accordance with the institutional animal welfare guidelines of Nanjing Medical University. All mice were maintained in specific pathogen-free (SPF) animal facilities with a 12 h day/12 h night cycle. Male BALB/c-nu mice (5 weeks old) were obtained from the Animal Research Center of Nanjing Medical University. To evaluate the effect of miR-876-5p on tumorigenesis and metastasis, 1 × 10^6^ CAL27 cells (0.2 mL) were suspended in Matrigel (BD) and were injected under the mice dorsa. When the tumors reached 3.0–5.0 mm in diameter (6 weeks old), the mice were randomly assigned into two groups (n = 7 each). Agomir-876-5p or negative control (NC) (RiboBio, Guangzhou, China) was directly injected into the implanted tumor at the dose of 5 nmol (in 50 µL of PBS) per mouse every 3 days for six times. Tumor diameter (mm) was measured every 2 days for 3 weeks, and tumor volume (V) was monitored by measuring the length (L) and width (W) with Vernier calipers and calculated with the formula V = (L × W^2^)/2. The tumor specimens were fixed in 4% PFA solution for 12–24 h and paraffin embedded for further analysis. For hepatic dissemination assays, HN6 cells (1 × 10^6^/mL) transfected with agomir-876-5p or NC (RiboBio, Guangzhou, China) were suspended in 200 µL of PBS for each mouse (3 per group). The cells were injected into nude mice through the lateral tail vein. After 3 weeks, the mice were killed, and the liver tissue was dissected, fixed with 4% PFA solution and embedded in paraffin. All sections were stained in hematoxylin and eosin (H and E) to analyze the tumor cells, and the number of micrometastases on the liver surface was calculated.

### Statistical analysis

SPSS18.0 software was used for statistical analysis. All data were presented as mean ± standard deviation (SD) of three independent experiments and evaluated using the Student’s t-test. One-way analysis of variance (ANOVA) was used for comparison, and P < 0.05 was considered statistically significant (*P < 0.05, **P < 0.01, and ***P < 0.001).

## Results

### The expression of miR-876-5p and vimentin in HNSCC cell lines and HNSCC tissues

**Fig. 1:**
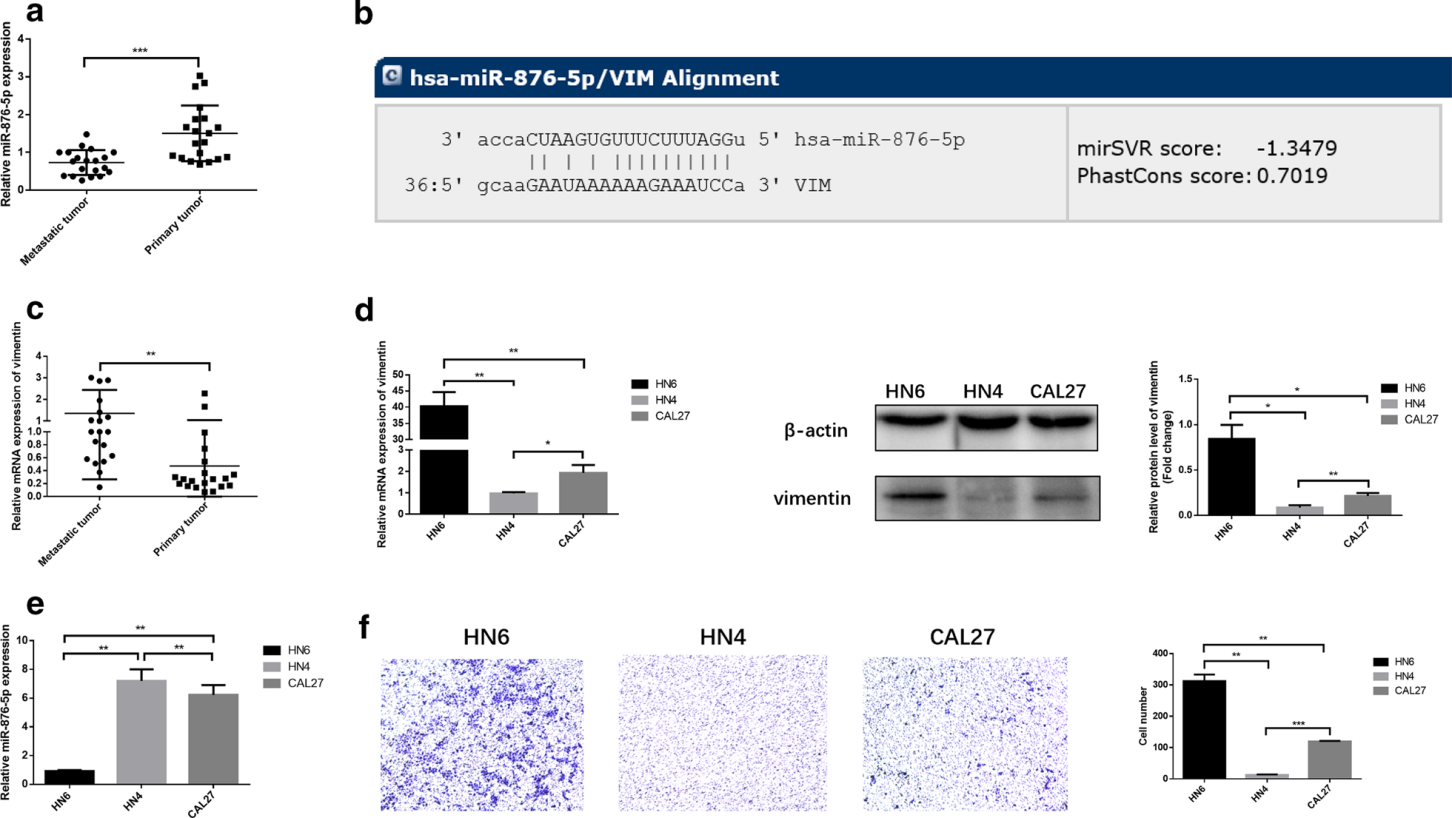
The expression of miR-876-5p and vimentin expression in HNSCC cell lines and HNSCC tissues. **a** The relative miR-876-5p mRNA expression in HNSCC tissues with associated regional nodal metastases and without metastatic primary tumors in situ. **b** A potential miR-876-5p binding site in the 3′-UTR of vimentin was predicted using TargetScan7.1. **c** The relative vimentin mRNA expression levels in metastatic HNSCC tissues and non-metastatic HNSCC tissues. **d**, **e** The miR-876-5p and vimentin expression level in HN4, HN6 and CAL27 cells. **f** Differences in the invasiveness of HN4, HN6 and CAL27 cells. The images were captured at × 200 magnification. *P < 0.05, **P < 0.01, ***P < 0.001

To evaluate the impact of miR-876-5p on HNSCC metastasis and progression, we performed quantitative PCR analysis to compare the miR-876-5p expression in metastatic HNSCC and non-metastatic HNSCC. In 20 cases of non-metastatic HNSCC tissues and HNSCC tissues with lymph node metastasis (Table 1), our results revealed that miR-876-5p expression was significantly lower in HNSCC tissues with lymph node metastasis than that in HNSCC without metastatic primary tumors (Fig. 1a). We then searched for potential miR-876-5p targets using TargetScan7.1 (http://www.targetscan.org) and found that the 3′-UTR of vimentin contains a potential miR-876-5p binding site (Fig. 1b). It has been reported that vimentin is usually expressed in normal mesenchymal cells and is critical for malignant cell invasiveness [26]. In HNSCC samples, we found that the vimentin mRNA expression levels were markedly upregulated in metastatic HNSCC tissues compared with non-metastatic HNSCC tissues, implying the complementary relationship between miR-876-5p and vimentin (Fig. 1c). Next, we examined the relative levels of vimentin and miR-876-5p in HN4, HN6 and CAL27 cells. High vimentin expression levels were detected in HN6 cells, but much lower vimentin levels were detected in HN4 and CAL27 cells (Fig. 1d). However, high miR-876-5p transcript levels were verified in HN4 and CAL27 cells, while much lower miR-876-5p levels were found in HN6 cells (Fig. 1e). Therefore, miR-876-5p expression levels may be negatively associated with vimentin levels in these HNSCC cells. Then, we performed transwell assays to evaluate the invasive ability of HNSCC cells. The results revealed that HN6 had the greatest invasiveness, HN4 had the worst, and CAL27 was between these two (Fig. 1f). Thus, we speculated that as a mesenchymal marker, vimentin may play a crucial role in HNSCC cell invasiveness and miR-876-5p may target vimentin to regulate HNSCC cell invasion ability.

### MiR-876-5p significantly inhibits cell migration and invasion in HNSCC cell lines

**Fig. 2:**
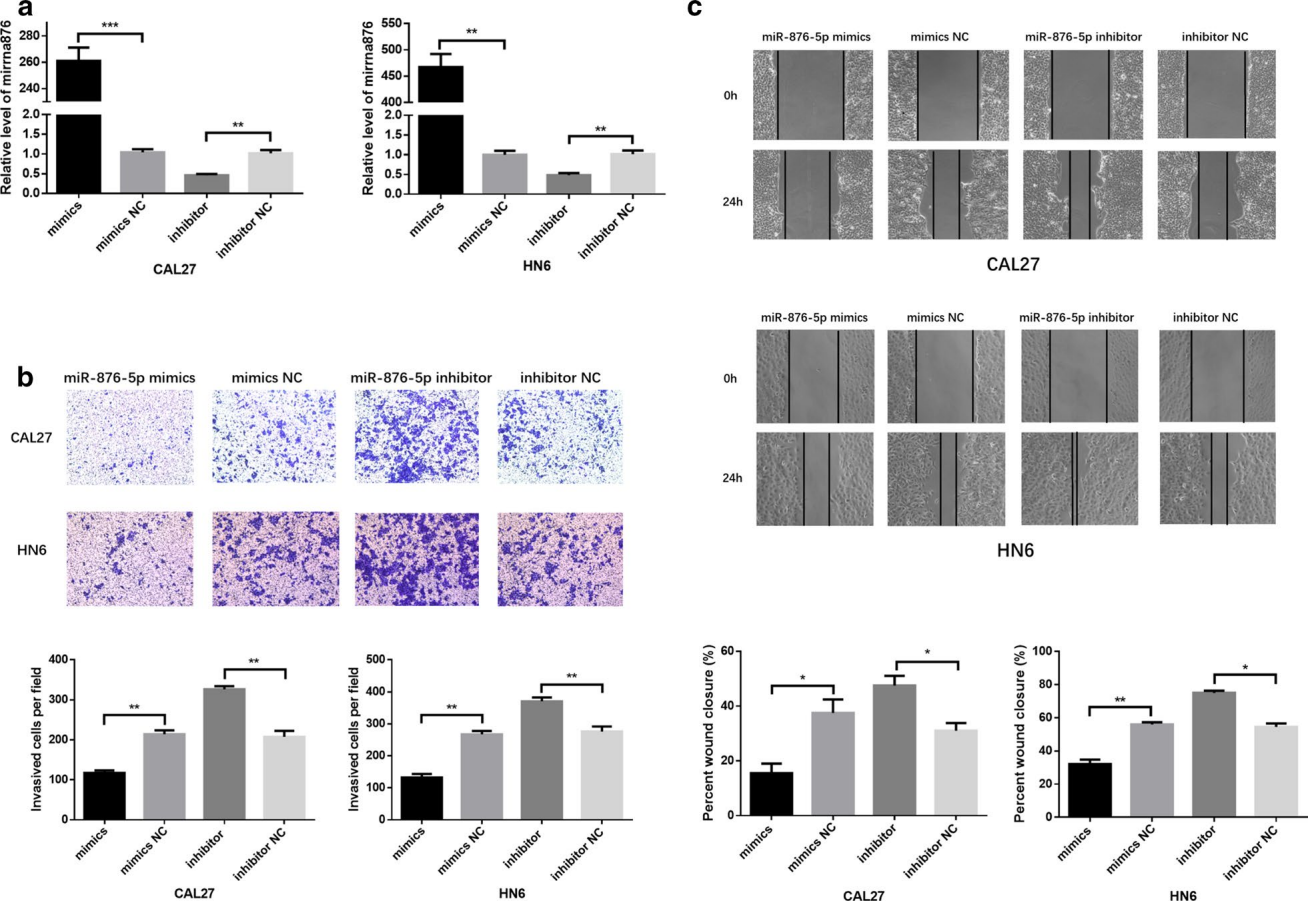
MiR-876-5p significantly inhibits cell migration and invasion in HNSCC cell lines. **a** MiR-876-5p expression after transfection. **b**, **c** The effects of miR-876-5p mimics and inhibitors on HNSCC cell invasion and migration. The wound area of scratch assays images were captured at ×40 magnification. The images of transwell assays were captured at ×200 magnification. *P < 0.05, **P < 0.01, ***P < 0.001

To evaluate the effect of miR-876-5p on invasion and migration in HNSCC, we transfected HN6 and CAL27 cells with miR-876-5p mimics or inhibitors. Transwell and scratch assays were utilized to verify the alteration of cell invasion and migration. Following transfection, increased miR-876-5p expression in HNSCC cells was confirmed by quantitative RT-PCR (Fig. 2a). In the miR-876-5p mimics group, miR-876-5p was overexpressed, while the invasion and migration activity of HNSCC was decreased markedly (Fig. 2b, c). On the other hand, compared with negative control cells, cells with miR-876-5p knocked down presented improved cell invasion and migration (Fig. 2b, c). Our data indicated that miR-876-5p inhibits the metastatic potential of HNSCC cells.

Moreover, to determine the alteration of cell proliferation, CCK-8 assays and flow cytometry were performed in HNSCC cells. Compared with the negative control, the miR-876-5p mimics or inhibitors did not substantially affect HNSCC cell proliferation (Additional file 1: Figure S1A, B).

### Vimentin is a direct target of miR-876-5p in HNSCC

**Fig. 3:**
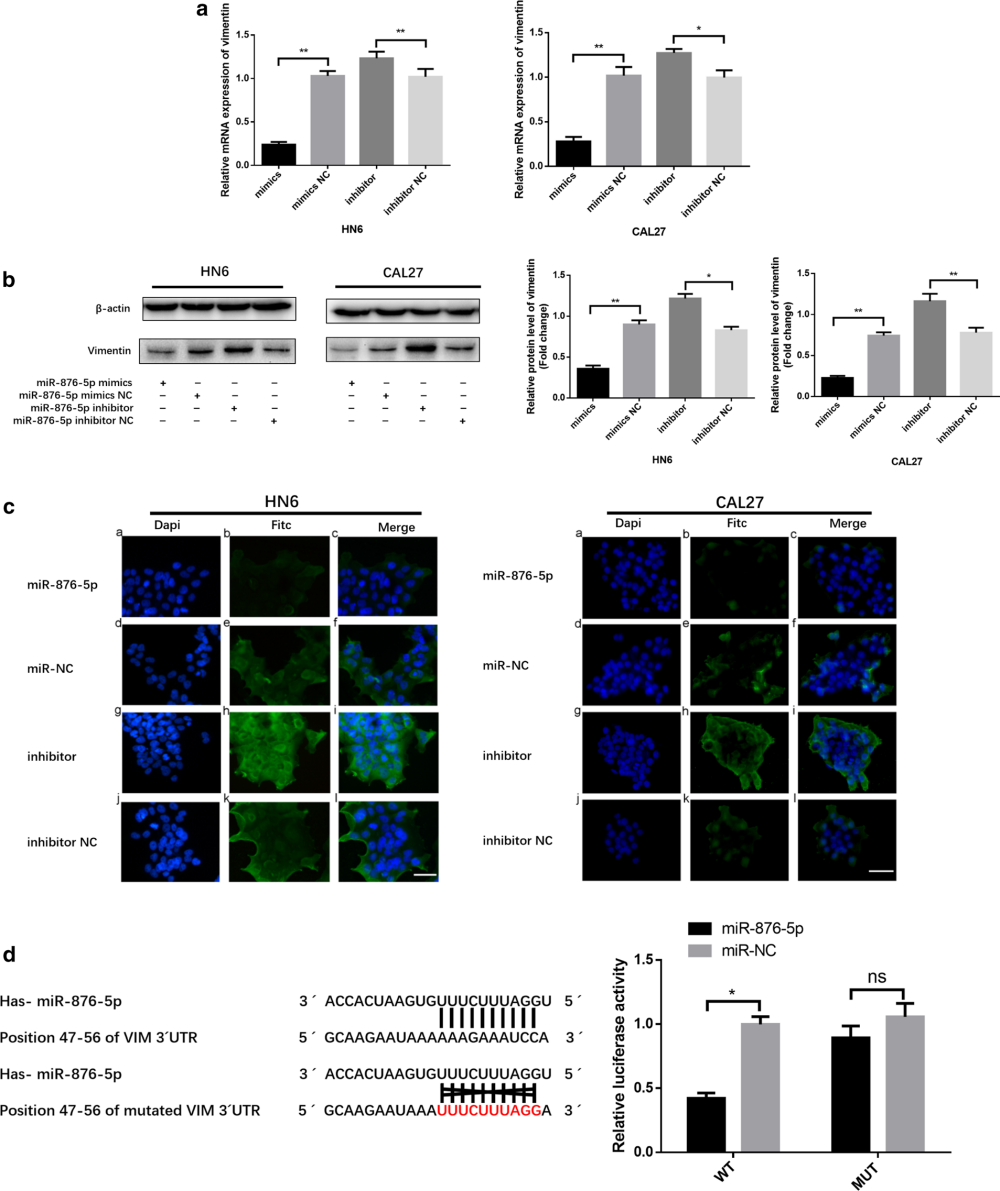
Vimentin is a direct target of miR-876-5p in HNSCC. **a**, **b** The relative vimentin mRNA and protein expression levels were detected by qRT-PCR and western blot assays. **c** Immunofluorescence assays were used to analyze vimentin expression in HNSCC cells. Scale bar,50 μm. **d** MiR-876-5p expression inhibits wild-type but not mutant vimentin 3′-UTR reporter activity. *P < 0.05, **P < 0.01, ***P < 0.001

To further examine the potential negative regulatory effect of miR-876-5p on vimentin, we transiently transfected HN6 and CAL27 cells with the miR-876-5p mimics or negative control. Vimentin mRNA expression in miR-876-5p mimic-transfected HNSCC cells was only 27% and 21% of that in cells transfected with negative control, respectively (Fig. 3a). Next, vimentin protein expression was quantified by western blot analysis after miR-876-5p transfection. The vimentin expression level was decreased in the miR-876-5p mimics group. In contrast, after cells were transfected with miR-876-5p inhibitors, vimentin protein expression was upregulated (Fig. 3b). Moreover, we investigated vimentin expression in HNSCC cells by immunofluorescence; As expected, vimentin expression was deregulated in the miR-876-5p mimics group and was upregulated after transfection with the miR-876-5p inhibitors (Fig. 3c). Therefore, our data suggested that miR-876-5p may limit the metastatic potential of HNSCC cells by inhibiting vimentin.

To investigate whether miR-876-5p binds the 3′-UTR of target mRNA, we generated a luciferase reporter wild-type vector that contained the vimentin 3′-UTR with the putative miR-876-5p binding sites. Correspondingly, we constructed a mutant reporter vector that contained the vimentin 3′-UTR with a mutation at the putative miR-876-5p binding site. Co-transfection experiments revealed that miR-876-5p downregulated the luciferase activity of the wild-type vector by 50%, but this decrease was not present when the mutant vector was transfected (Fig. 3d). Taken together, these results indicate that vimentin is a direct, downstream target of miR-876-5p.

### Vimentin overexpression promotes HNSCC cell migration and invasion

**Fig. 4:**
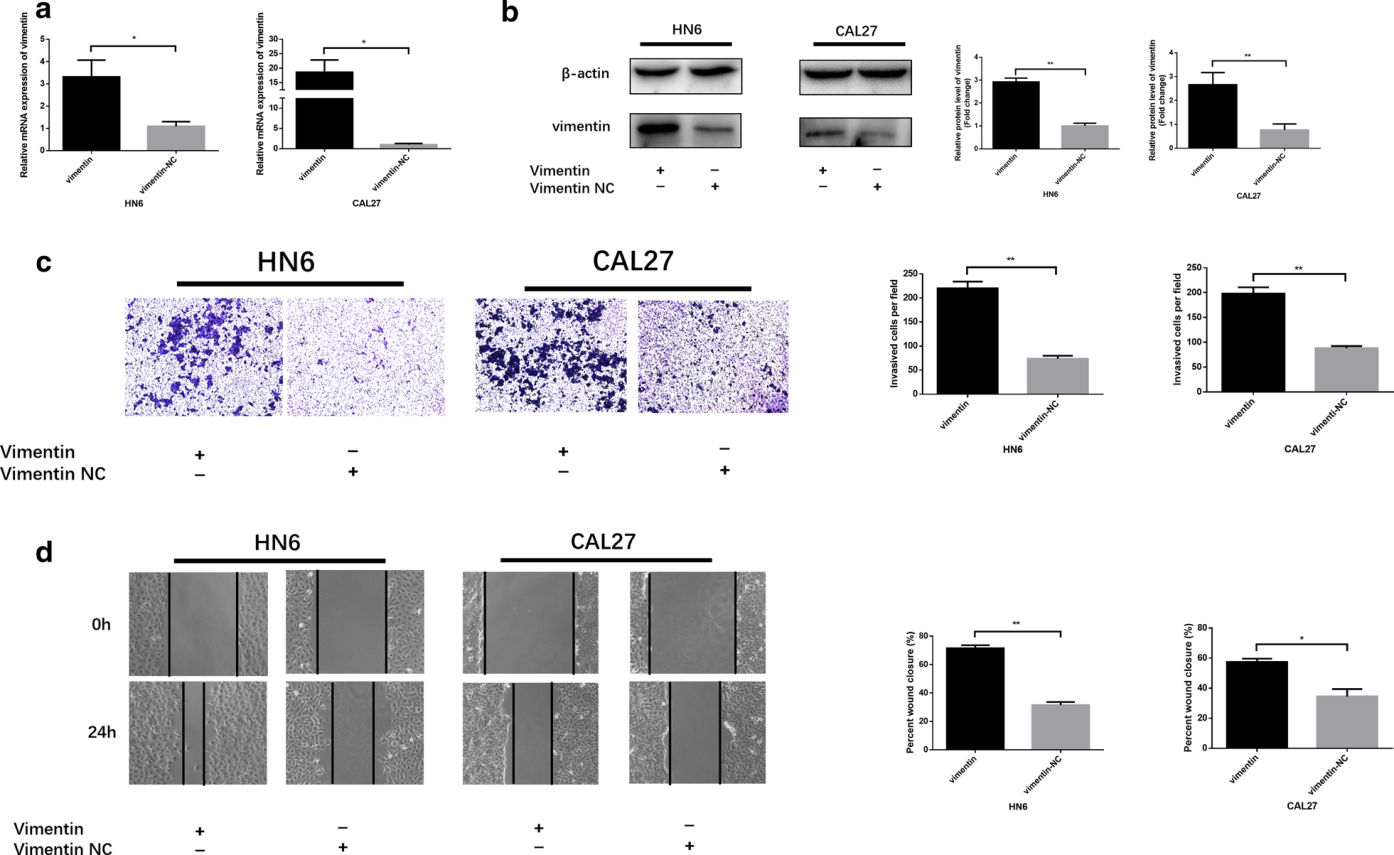
Vimentin overexpression promotes HNSCC cell migration and invasion. **a**, **b** Vimentin mRNA and protein expression after transfection. **c**, **d** The effects of vimentin overexpression on HNSCC cell invasion and migration ability. The wound area of scratch assays images were captured at ×40 magnification. The images of transwell assays were captured at ×200 magnification. *P < 0.05, **P < 0.01, ***P < 0.001

HN6 and CAL27 cells were transfected with special vimentin-overexpressing plasmids using Lipofectamine 2000. After transfection, the mRNA and protein expression level were obviously increased according to the qRT-PCR and western blot analysis (Fig. 4a, b). By using scratch and transwell assays, we evaluated whether vimentin upregulated the migratory and invasive ability of HNSCC cells. As expected, vimentin overexpression significantly induced HNSCC cell invasion abilities (Fig. 4c). According to the results in Fig. 4d, HN6 and CAL27 cell migratory abilities were increased significantly in the vimentin-overexpressing group. These results revealed that vimentin significantly regulates HNSCC cell invasion and migration.

### Vimentin is a critical mediator of miR-876-5p in HNSCC cells

**Fig. 5:**
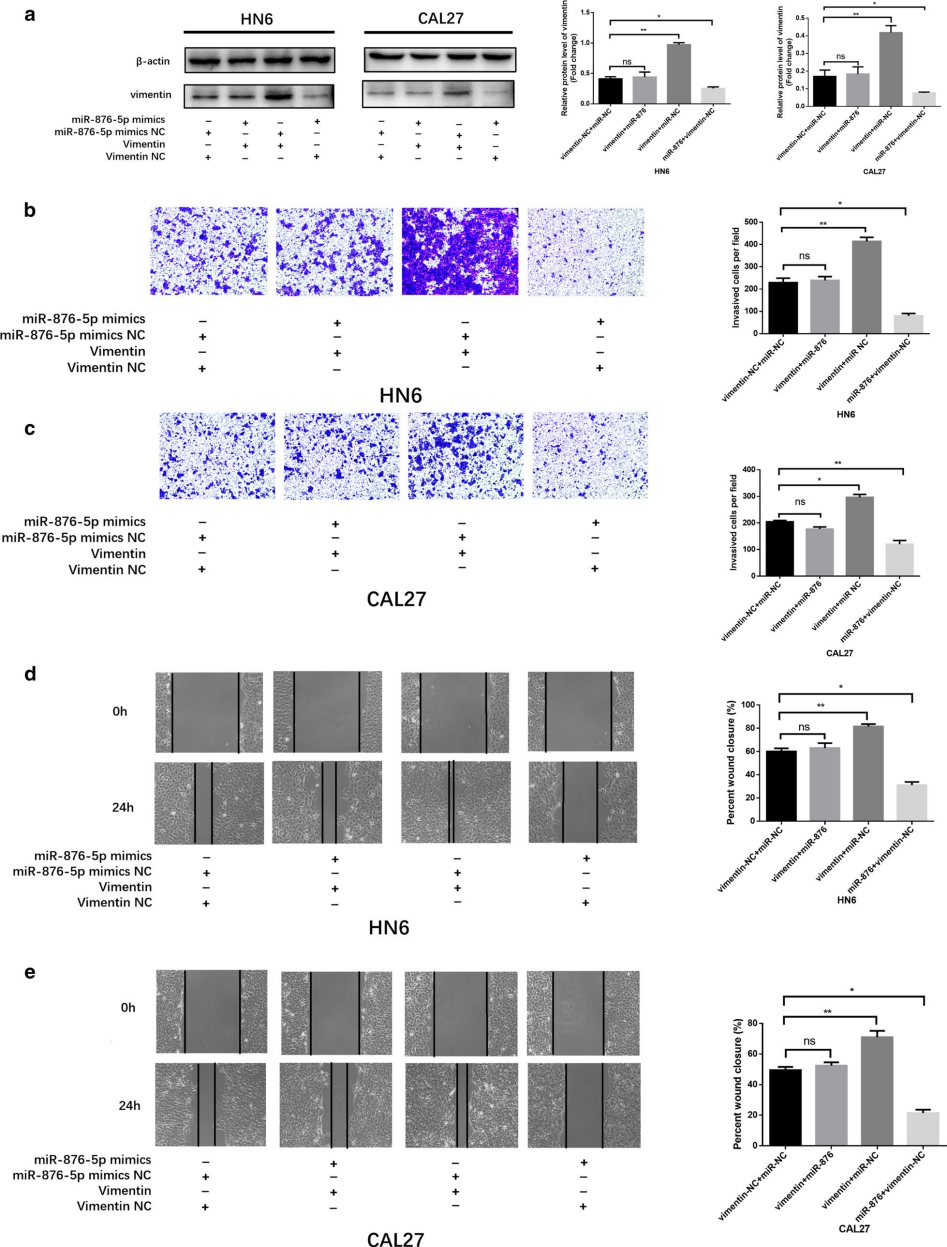
Vimentin is a critical mediator of miR-876-5p in HNSCC cells. **a** Co-transfection of miR-876-5p mimics with vimentin overexpression restored vimentin expression to the original level in HNSCC cells. **b**–**e** MiR-876-5p mimics decreased HNSCC cell invasion and migration, whereas co-transfection with EX-vimentin-M02 negated these changes. The wound area of scratch assays images were captured at ×40 magnification. The images of transwell assays were captured at ×200 magnification. *P < 0.05, **P < 0.01, ***P < 0.001

To further investigate whether miR-876-5p targets vimentin to regulate HNSCC cell invasion and migration, we divided into four groups: miR-876-5p-NC co-transfection with vimentin-NC, miR-876-5p mimics co-transfection with vimentin overexpression, miR-876-5p-NC together with vimentin overexpression, and miR-876-5p mimics together with vimentin-NC. As shown in Fig. 5a, transfection of vimentin plus miR-876-5p mimics significantly attenuated vimentin expression. Transwell assays revealed that cell invasiveness was higher in the over expressed vimentin group and lower in the miR-876 mimics group than that in the control group. No significant difference in the number of invasive cells per field was observed between the miR-876-5p mimics with the overexpression vimentin group and control group (Fig. 5b, c). Similarly, in scratch assays, vimentin increased HNSCC cell migratory ability, whereas miR-876-5p mimics blocked this change (Fig. 5d, e). Hence, our findings demonstrated that miR-876-5p regulates HNSCC cell invasion and migration via targeting vimentin.

### MiR-876-5p inhibits vimentin expression and suppresses the distal metastasis of HNSCC cells in vivo

**Fig. 6:**
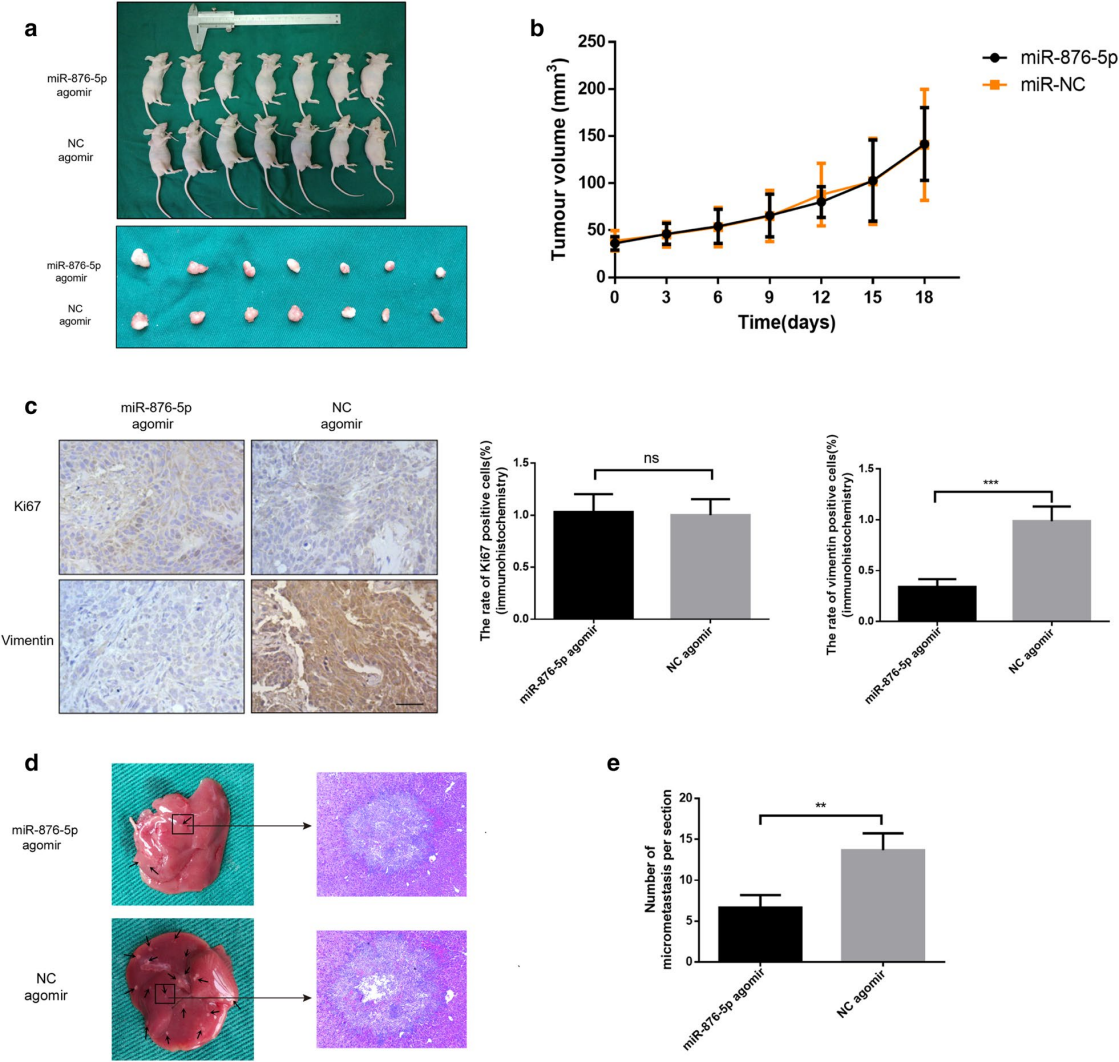
MiR-876-5p inhibits vimentin expression and suppresses hepatic metastasis of HNSCC cells in vivo. **a**, **b** Tumor volume was not significantly different between the agomir-876-5p and negative control groups. Each group contains seven nude mice. **c** Histological and immunohistochemical analysis of Ki-67 and vimentin. Scale bar, 50 μm. **d** Representative histological images show tumor metastases in the liver of nude mice. **e** The number of micrometastases on the liver surface was calculated. Each group contains three nude mice. *P < 0.05, **P < 0.01, ***P < 0.001

Since miR-876-5p substantially suppressed HNSCC cell migration and invasion in vitro, we further investigated the biological role of miR-876-5p in vivo. After incubating with agomir-876-5p, 2 × 10^6^ CAL27 cells were injected into the right dorsal subcutaneous tissue of nude mice. Two weeks after inoculation, agomir-876-5p was injected into the tumor tissues once every 3 days (5 nmol/mice). After 3 weeks of treatment, all mice were sacrificed, and the tumors extracted from the mice were presented in Fig. 6a, b. We found that the volume of the tumors formed by miR-876-5p-overexpressing CAL27 cells was not significantly different from that of the tumors formed by NC-expressing cells. IHC staining showed that the expression of the metastasis-related protein vimentin was upregulated, but proliferation-related protein Ki-67 [27] was not decreased in the tissue samples from the miR-876-5p-transfected nude mice. This data demonstrated that miR-876-5p has no significant effect on tumor proliferation in vivo (Fig. 6c).

To further determine the effect of miR-876-5p overexpression on HNSCC cell metastasis in vivo, we performed the distal dissemination assays using a nude mouse model. BALB/c nude mice were injected with agomir-876-5p or NC transfected cells via the tail vein (group 2, n = 3). At 24 days post injection, all mice were sacrificed. For the distal dissemination assay, the liver tissues were removed and subjected to histological examination. As expected, miR-876-5p substantially reduced the number of metastatic HNSCC cell clumps on the surface of the liver (Fig. 6d). These data suggest that miR-876-5p suppresses HNSCC cell metastasis in vivo.

## Discussion

During HNSCC initiation and progression, miRNAs can modulate diverse biological processes, acting as tumor oncogenes or suppressors [28, 29]. An increasing amount of evidence suggests that some miRNAs strongly regulate the metastatic and invasive ability of tumorigenic cells [30–32]. Hence, understanding abnormal miRNA expression might promote our understanding of HNSCC tumorigenesis and provide critical information for the identification of novel markers and drug targets to treat HNSCC.

Due to the increased incidence of HNSCC in both developed and developing country, the potential molecular mechanisms of HNSCC have received more attention [33–35]. According to previous studies, more abnormally expressed miRNAs have been found in HNSCC. For example, miR-30a and miR-934 have been reported to be significantly upregulated in alcohol-associated HNSCC [36]. Besides, the miR-124 expression level was 4.59-fold lower in HNSCC tissues than in normal tissues [37]. Also, some researchers found that miR-422a was significantly decreased in oropharynx tumors from patients who experienced early loco-regional recurrence [38]. Furthermore, miR-145-5p and miR-145-3p expression levels were significantly down-regulated in HNSCC tissues and cell lines [39]. In our study, we found that miR-876-5p is down-regulated in HNSCC cell lines and tissues. Transfection of miR-876-5p significantly inhibited cell migration and invasion, indicating the anti-tumor effect in HNSCC.

EMT is a biological process in which a non-motile epithelial cell is converted into a mesenchymal phenotype accompanied by increased invasiveness [40]. One of the main characteristics of cancer cells undergoing EMT is the enhanced expression of mesenchymal markers, such as vimentin [18, 41, 42]. Some studies have shown that high vimentin expression is associated with enhanced HNSCC cell metastatic and invasion potential [23, 43]. A growing number of evidence has established that miRNAs affect cancer development by regulating vimentin. In HNSCC, miR-375 indirectly regulates vimentin mRNA levels, which is important for HNSCC invasion [44]. In colorectal cancer, miR-378 may function as a tumor suppressor and plays an important role in inhibiting tumor growth and invasion by targeting vimentin [8]. In addition, miR-30a inhibits vimentin expression, which may serve as a tumor biomarker for predicting breast cancer outcome and assist in the development of a potential therapeutic target for this disease [45]. By using bio-informatics analysis, we found that vimentin could be a direct target of miR-876-5p in the process of downregulating HNSCC metastasis. The data in our study has provided the first instance that investigates the involvement of miR-876-5p in human HNSCC.

In this study, all our data indicated that miR-876-5p might be a novel tumor suppressor which impacted HNSCC metastasis. To further clarify the alteration of cell proliferation, CCK-8 assays and flow cytometry were performed in the experiment. We found that the cell proliferation ability was not significantly changed between cells transfected with the miR-876-5p mimics and negative control. In addition, in vivo studies demonstrated that subcutaneous tumor volume was not substantially reduced with miR-876-5p overexpression. Meanwhile, the IHC statistical scoring stained with Ki-67 revealed no marked change between the tumors from cells injected with agomir-876-5p and those from cells injected with NC. Therefore, we hypothesized that miR-876-5p may not serve as a novel marker in cell proliferation in HNSCC.

To better understand the potential molecular mechanism of the antitumor effects of miR-876-5p in HNSCC, we used TargetScan (http://www.targetscan.org) to search for the potential target genes of miR-876-5p. We found that the vimentin 3′-UTR sequence was predicted to harbor one highly conserved miR-876-5p binding site. In the present investigation, we demonstrated that miR-876-5p was closely related to HNSCC cell invasiveness. Through luciferase reporter assays, we confirmed that vimentin was a direct downstream target for miR-876-5p. Moreover, miR-876-5p overexpression significantly inhibited HNSCC cell migration and invasion in vitro and in vivo. Interestingly, recovery of the vimentin expression level improved HNSCC cell migration and invasion. These results supported the use of miR-876-5p as a characteristic prognosis marker and potential predictor of cervical lymph node metastasis in HNSCC.

## Conclusions

Taken together, our data suggested a significant role of miR-876-5p in inhibiting HNSCC invasiveness and metastasis. Co-transfection with expression vector of vimentin and miR-876-5p effectively reduced the expression of vimentin and reversed its oncogenic property in HNSCC cells. The finding that miR-876-5p downregulates vimentin expression might provide a novel diagnostic marker and therapeutic target for HNSCC treatment.

## Additional file

**Additional file 1: Figure S1.** MiR-876-5p had no significant effect on proliferation in HNSCC cells. **(A)** CCK-8 growth curves of CAL27 and HN6 cell lines after transfection. The effects on proliferation of HNSCC cells was not significantly different between the miR-876-5p mimics and inhibitors. **(B)** The cell-cycle analysis by flow cytometry revealed no significantly different in S phase between the miR-876-5p mimics and inhibitors. *P<0.05, **P<0.01, ***P<0.001.

## Abbreviations

HNSCC: head and neck squamous cell carcinoma
EMT: epithelial–mesenchymal transition
CCK-8: cell counting kit-8
IHC: immunohistochemistry
HE: hematoxylin and eosin (H and E)
IF: immunofluorescence staining
BSA: bovine serum albumin
PVDF: polyvinylidene fluorid
