# Supplementary figures and images for: MiR-876-5p modulates head and neck squamous cell carcinoma metastasis and invasion by targeting vimentin
Source: Cancer Cell Int. 2018 Aug 28;18:121. doi: 10.1186/s12935-018-0619-7 (PMC6114268; doi:10.1186/s12935-018-0619-7)

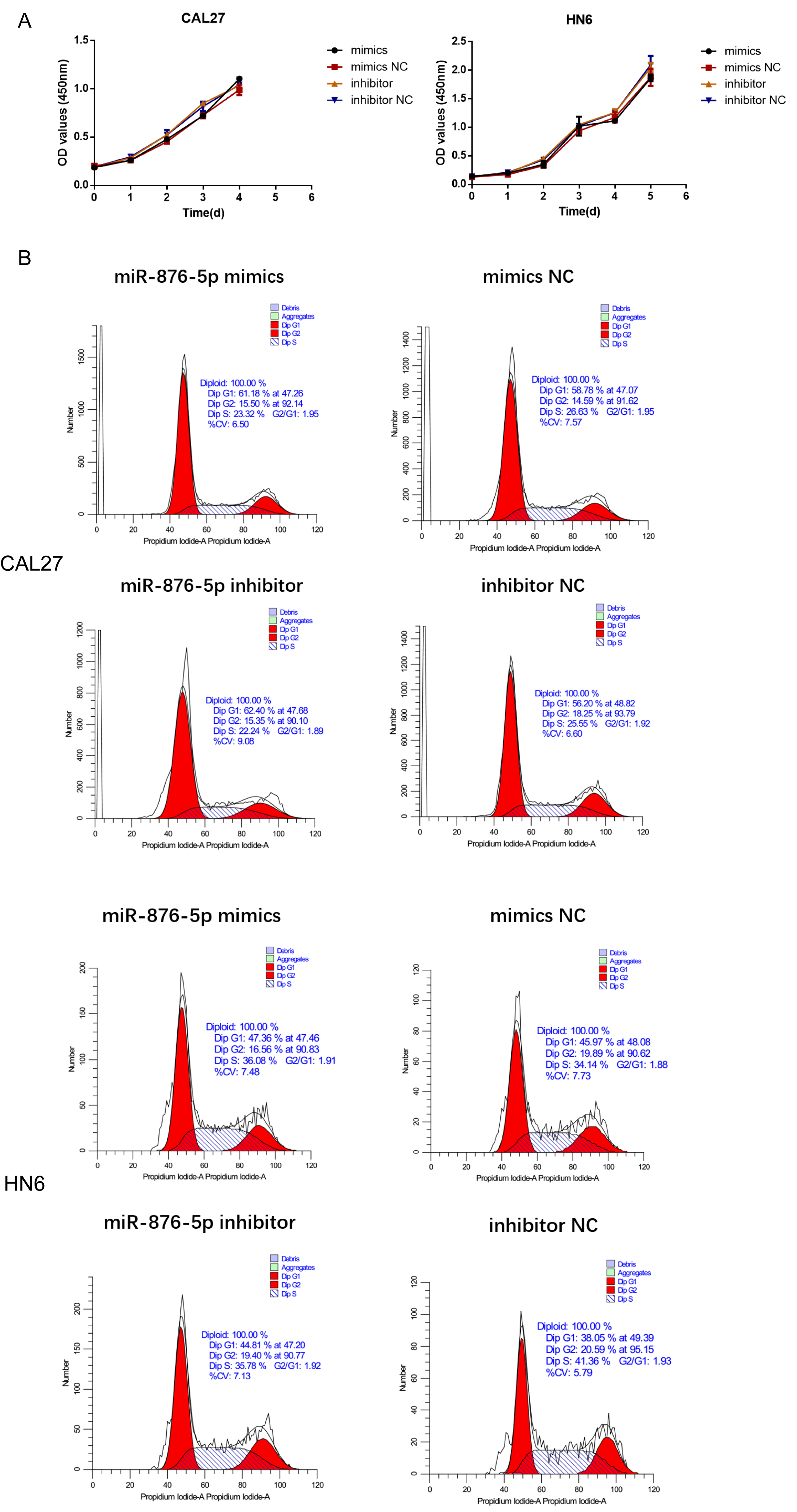

Supplement: Supplementary file 1 — Additional file 1: Figure S1. MiR-876-5p had no significant effect on proliferation in HNSCC cells. (A) CCK-8 growth curves of CAL27 and HN6 cell lines after transfection. The effects on proliferation of HNSCC cells was not significantly different between the miR-876-5p mimics and inhibitors. (B) The cell-cycle analysis by flow cytometry revealed no significantly different in S phase between the miR-876-5p mimics and inhibitors. *P<0.05, **P<0.01, ***P<0.001. [file 12935_2018_619_MOESM1_ESM.tif]
